# Fight against Epidemics

**Published:** 1920-05-15

**Authors:** 


**THE PUBLIC WELL-BEING**—(*continued*).

### FIGHT AGAINST EPIDEMICS.

### Scheme to Prevent Spread from the East.

The typhus epidemic in Poland closely affects the material interests of many nations in and outside Europe. Measures are needed for their own safety against the spread of an epidemic disease which becomes most formidable among populations undergoing social disturbances, and, in consequence of war conditions, suffering from malnutrition. The settlement of Europe may be incalculably affected by the continuance and extension of these serious epidemics—a matter in which the whole civilised world is concerned.

A typhus epidemic has raged in Poland for several years; in 1916 there were 34,538 cases, in 1917 43,840, in 1919 231,206, in January and February 1920, 46,500. The cases which have occurred in the vast Eastern territories at present administered by Poland are not included in this figure. The epidemic is spread by the prisoners of war and the emigrants returning from Russia, where the disease has its permanent centre of infection. (Between the end of 1918 and January 1920 the number of persons coming from the East who have passed through or settled down on Polish territory reached 1,279,692).

The Polish Government has spared itself no sacrifice in combating this plague; the credits which it has allotted for fighting the disease absorb 1.5 per cent. of the total Polish budget, including war expenditure. It would be difficult to find in the budget of any other state so high a proportion of money apportioned for the fighting of disease.

The International Red Cross, the Y.M.C.A. and other similar organisations were the first to come to the Polish Government's assistance, but all their combined efforts have not proved sufficient to stop the spread of the epidemic westwards.

The Council of the League of Nations invited the Health Conference, which was summoned to draw up proposals for the establishment of a permanent health organisation, to take upon itself in advance the duty that would devolve upon the permanent organisation, and to interest itself at once

**THE PUBLIC WELL-BEING**—(*continued*).

in the typhus epidemic in Poland. The Health Conference, summoned on April 13 in London by the British Health Minister, came to the conclusion that the League of Nations was the only organisation capable of adequately undertaking the fight against the Polish epidemics; a fight which demands resources and endeavours beyond the power of any single Government. In view of the findings of this Conference, the organisation and increase of preventive measures against the spread of typhus is an urgent duty incumbent upon all members of the League.

The Conference recognised that the measures adopted by the Polish Government were efficacious, and accepted the Polish scheme of organisation for the fight against the spread of epidemics coming from the East. Under this scheme it would be absolutely necessary to double the number of Quarantine Stations in Poland, which form the sanitary cordon, and to bring the number up to twenty-four; to increase the number of beds in the hospitals to 30,000 (at present there are only 13,077); to form a thousand flying sanitary columns, and to take in hand the distribution of food-stuffs and clothes and to organise transport facilities.

The scheme gives a detailed description of the sanitary material required. It should be carried out by two commissioners appointed by the League of Nations: a chief commissioner in charge of purchases and the collection and despatch of the material into Poland, and a medical commissioner in charge of the collection and despatch of sanitary units and of the medical personnel. The distribution and employment of the personnel and of the material supplied by the League of Nations would be decided upon with the advice of the proper Polish authorities.

The Council of the League of Nations will examine this proposal at its meeting in Rome this week.

